# Mutual Use of Trail-Following Chemical Cues by a Termite Host and Its Inquiline

**DOI:** 10.1371/journal.pone.0085315

**Published:** 2014-01-21

**Authors:** Paulo Fellipe Cristaldo, Og DeSouza, Jana Krasulová, Anna Jirošová, Kateřina Kutalová, Eraldo Rodrigues Lima, Jan Šobotník, David Sillam-Dussès

**Affiliations:** 1 Departamento de Entomologia, Universidade Federal de Viçosa, Minas Gerais, Brazil; 2 Institute of Organic Chemistry and Biochemistry, Prague, Czech Republic; 3 Faculty of Science, Charles University in Prague, Prague, Czech Republic; 4 Faculty of Forestry and Wood Sciences, Czech University of Life Sciences, Prague, Czech Republic; 5 Institut de Recherche pour le Développement, Unité Mixte de Recherche 211 Biogéochimie et Ecologie des Milieux Continentaux, Interactions Biologiques dans les Sols, Bondy, France; 6 Laboratoire d'Ethologie Expérimentale et Comparée, Equipe d'accueil 4443, Université Paris 13, Sorbonne Paris Cité, Villetaneuse, France; University of Freiburg, Germany

## Abstract

Termite nests are often secondarily inhabited by other termite species ( = inquilines) that cohabit with the host. To understand this association, we studied the trail-following behaviour in two Neotropical species, *Constrictotermes cyphergaster* (Termitidae: Nasutitermitinae) and its obligatory inquiline, *Inquilinitermes microcerus* (Termitidae: Termitinae). Using behavioural experiments and chemical analyses, we determined that the trail-following pheromone of *C. cyphergaster* is made of neocembrene and (3Z,6Z,8E)-dodeca-3,6,8-trien-1-ol. Although no specific compound was identified in *I. microcerus*, workers were able to follow the above compounds in behavioural bioassays. Interestingly, in choice tests, *C. cyphergaster* prefers conspecific over heterospecific trails while *I. microcerus* shows the converse behaviour. In no-choice tests with whole body extracts, *C. cyphergaster* showed no preference for, while *I. microcerus* clearly avoided heterospecific trails. This seems to agree with the hypothesis that trail-following pheromones may shape the cohabitation of *C. cyphergaster* and *I. microcerus* and reinforce the idea that their cohabitation is based on conflict-avoiding strategies.

## Introduction

A wide variety of species adopt the strategy to live in close association to each other, establishing symbiotic interactions (see e.g. [1]–[4]). Inquilinism stands among the most specialized forms of symbioses: more than symbiotically interacting in the open space, inquilines cohabit with their host in the nest which these latter have built to house their own relatives [5]. Fundamentally, inquilines are equivalent to better known symbionts, such as gut-inhabiting bacteria, but differ in being naked-eye observable and easily manipulated in lab assays. The ubiquity of inquilinism is impressive and examples include red-headed woodpeckers, cuckoos and cowbirds [6], [7], salamanders [8], and especially social insects (see [9]–[14]). While existing in virtually all known social insects, subtle differences can be observed between Hymenoptera and Isoptera inquilines. In Hymenopterans, inquilines (or “social parasites”) live in close contact with their host as brood parasites [15], whereas in termites, inquilines tend to establish themselves apart from their hosts within the nest [16]. In termites the term “inquilinism” is reserved for heterospecific termite-termite cohabitation [14]. Low frequency of direct contact between termite inquilines and their hosts by no means precludes the need to negotiate cohabitation. By occupying a space originally built for the host's nestmates, feeding on the lining of the nest walls, or stealing stored products [17]–[20], inquilines most certainly inflict costs to their hosts. Chances of occasional encounters are increased by the fact that cohabitation may take place in volumes as small as 13 litres [14], with inquilines inhabiting the “heart of the hive” and being outnumbered by their host [5]. All this would enhance selective pressures for defensive strategies on the part of the host with consequent development of deceiving strategies on the part of inquilines, establishing arms races likely similar to those observed for cuckoos *versus* hosts in birds and in other social insects [21].

Because termite defence is carried out by blind individuals, it is conceivable to think that both sets of strategies would rely markedly on chemical cues. After all, such cues allow social insects to behave altruistically towards nestmates and discourage the presence of intruders in their society [9]. One of these signals is the trail-following pheromone, which in termites presents surprisingly low chemical diversity, with only 9 active compounds (alcohols, aldehydes, ketone, and hydrocarbons) identified so far [22]–[24]. This low complexity is further reduced by phylogenetic constraints: in the basal termites (Mastotermitidae, Archotermopsidae, Stolotermitidae), trail-following pheromones are composed of C14 or C18 aldehydes, while in all higher termites, trail pheromones comprise C12 alcohols and/or hydrocarbon neocembrene [22], [23]. The only exception is *Glossotermes*, member of the phylogenetically transitional family Serritermitidae [25], whose trail-following pheromone has C19 ketone [24]. Single component trail-following pheromones have been identified in all studied termite species except *Prorhinotermes simplex* (Rhinotermitidae), *Amitermes evuncifer* (Termitidae: Termitinae) and several Nasutitermitinae (Termitidae), in which the pheromone always consists of neocembrene and (3*Z*,6*Z*,8*E*)-dodeca-3,6,8-trien-1-ol [26]–[28].

Such a low chemical diversity coupled with high phylogenetic similarity makes trail-following pheromones likely candidates for deceiving strategies on the part of inquilines. After all, manipulation of host-inquiline communication by inquilines would not need new physiologic routes to produce specific compounds.

To the best of our knowledge, no attempt to disentangle host-inquiline communication in termites has been made yet and the mechanisms of cohabitation between them remain enigmatic. Here, we investigated the hypothesis that trail-following pheromones may shape the association between host and inquilines in termites. As a model, we used *Constrictotermes cyphergaster* (Silvestri 1901) and its obligatory inquiline *Inquilitermes microcerus* (Silvestri 1901). *Constrictotermes cyphergaster* (Termitidae: Nasutitermitinae) is a common Neotropical termite species occurring in Brazil, Paraguay, Bolivia, and Northern Argentina [17]. Workers leave the nest in columns and forage at night in the open air under the protection of soldiers [29], and feed predominantly on debris [30] and lichens [19] on the surface of tree barks. *Constrictotermes* spp. nests harbour many organisms but they are not known to commonly house termite inquilines other than *Inquilinitermes* spp. [17], [31]. Among these, *I. microcerus* (Termitidae: Termitinae) is known to live exclusively in *C. cyphergaster* nests, in galleries separated from their host's [14], feeding on a highly decomposed diet which may consist of the lining of the nest walls [20]. The colonies are restricted to certain portions of the nest, usually close to its core [32].

To accomplish our aims, we have *(i)* studied the nature of trail-following pheromones in *C. cyphergaster* and *I. microcerus*, and *(ii)* tested for mutual recognition of one another's trails. Gas chromatography coupled with mass spectrometry was used to inspect chemical composition of these pheromones. Additionally, behavioural assays evaluated *(i)* the orientation of workers on conspecific *vs.* heterospecific trails, and *(ii)* the possible use of the host trail-following pheromone by the inquiline (for avoidance or orientation) and the use of the inquiline trail-following pheromone by the host (for detection and elimination of the inquiline).

## Materials and Methods

### Ethics Statement

A permit for termite collecting was provided by IBAMA to ODS, PFC and JŠ (33094). An export permit was provided by CNPq-Brazil (001347/2012-8) and the import permits were provided by Division of Protection against Harmful Organism-Czech Republic (SRS 032901/2012 and 032904/2012). No specific permits were required for the described studies undertaken in the laboratory with a non-endangered or protected species.

### Definitions

We adopt here the same terminology used by [20]: the term “nest” denotes the physical structure built by termites and the term “colony” denotes the assemblage of individuals of a given species, living and cooperating within the nest. “Cohabitation” refers to the simultaneous occurence of colonies of different termite species within a given nest, without implication of reciprocal positive or negative influences.

### Study site, Collection and Maintenance

Arboreal nests of *C. cyphergaster* containing colonies of *I. microcerus* were sampled near Sete Lagoas town (19′27°S, 44′14°W; Minas Gerais, Brazil); the site belongs to a vegetational formation physiognomically, but not floristically, similar to savannas (“cerrado”). We collected altogether 13 colonies, of which seven were transported to Viçosa (Minas Gerais, Brazil) in July 2012 and large parts of the six other colonies were transported to Prague in September 2012. The work started in Viçosa, where the colonies were kept in ambient lab conditions (±26°C and low relative air humidity), while in Prague the fragments of colonies were kept inside plastic boxes at temperature ±27°C and low relative air humidity. *Constrictotermes cyphergaster* was allowed to forage on large trays where bark covered with algae, mosses and lichens served as food and pieces of cotton soaked with water served as the water source; *I. microcerus* was never observed outside its galleries.

### Anatomy of sternal glands

Ten workers of both species, from distinct colonies, were anesthetized on ice and immediately dissected, embedded into Spurr's resin^(™)^ following a well-established protocol, sectioned with a Reichert-Jung Ultracut Microtome^(™)^ and studied with a Carl-Zeiss Amplival^(™)^ optical microscope (for details see [33]). Size of sternal glands was measured by ImageJ software. Additionally, the worker's body size in both species was measured.

### Preparation of whole bodies and sternal glands extracts

Whole body extracts (WBE) were prepared from workers (100–400 per sample) submerged in hexane (approximately 10 µl/1 worker) and kept for 24 h at 4°C. After this extraction, a second wash was done with approximately 100 µl of hexane at laboratory temperature, and both washes were merged. Subsequently, the volume of the extract was reduced under the nitrogen flow and the equivalent *per* worker serving as a measure in the bioassays was calculated. Sternal glands extracts (SGE) were prepared from the 4th and 5th sternites dissected from cold-immobilized workers (50–100 glands per sample), submerged into hexane (10 µl/1 gland), extracted for 6 h at 4°C and afterwards merged to a second wash done with 100 µl of hexane at laboratory temperature. After being merged, both extracts (WBE and SGE) were stored at −18°C prior to use. WBE extracts were prepared using host and inquiline workers from three distinct nests (hence, three colonies of hosts and three colonies of their respective inquilines). Likewise, SGE extracts were prepared from another three nests. Each of those colonies have been used only once and extracts originated from them were tested independently as distinct replicates of the bioassays, as described below (“Behavioural experiments” section). Each replicate was comprised of an extract prepared with workers from a single colony.

### Pheromone Standards

Synthetic standard of (3*Z*,6*Z*,8*E*)-dodeca-3,6,8-trien-1-ol (dodecatrienol; RI 1528) was kindly provided by Ullrich Jahn (Institute of Organic Chemistry and Biochemistry, Czech Republic). (1*E*,5*E*,9*E*,12*R*)-1,5,9-Trimethyl-12-(1-methylethenyl)cyclotetradeca-1,5,9-triene (neocembrene; RI 1959) was isolated from tergal glands of *Nasutitermes voeltzkowi* female alates (for details see [34]).

### Chemical analyses

Chemical analyses were performed using comprehensive two-dimensional gas chromatography coupled with mass spectrometric detection (GC×GC/TOF-MS; Pegasus 3D, Leco, St. Joseph, MI, USA). The first dimension column was a non-polar ZB5-MS (30 m, id 0.25 mm, 0.25 µm phase thickness) and the second dimension column was a polar RTX-50 (2 m, id 0.1 mm, 0.1 µm phase). The temperature program for the first column was 50°C (1 min) to 320°C (4 min) at 8°C/min range; the secondary column temperature was set 10°C higher.

Samples were concentrated to approximately 10 µl and then 1 µl was injected in a splitless mode. Injector temperature was 220°C. Helium (flow rate, 1.0 ml/min) was used as a carrier gas. Modulation period was 4 s. TOF-MS detector conditions were as follow: ion source temperature 220°C, detector voltage 1,750 V, filament base voltage −70 V, acquisition rate 100 spectra/s. Redistilled hexane (Merck, for organic trace analysis) was used for extracts and standard solutions.

### Behavioral experiments

The following bioassays were performed in open-field on Whatman N° 1 filter paper discs (Ø 15 cm diameter) covered by a large Petri dish, in ambient lab temperature and under dimmed light. Hamilton syringes (10 µl) were used in order to lay down the scent trails (see below) onto the filter paper, in all used concentrations. A worker was deposited in a release chamber (a plastic vial 3 cm in diameter with a 2 mm wide opening) from which it was allowed to walk on the filter paper to follow the scent trail. For each worker tested, a new trail was laid down on a new filter paper. Travelled distances and specific behaviours were recorded for each termite, as described below. Bioassays were carried out independently with both host and inquiline cohabiting workers using extracts made from the respective species and colonies. Each bioassay involved ten workers of both species collected from three colonies drawn randomly from the six available nests.

#### Intraspecific trail-following

**Figure 1:**
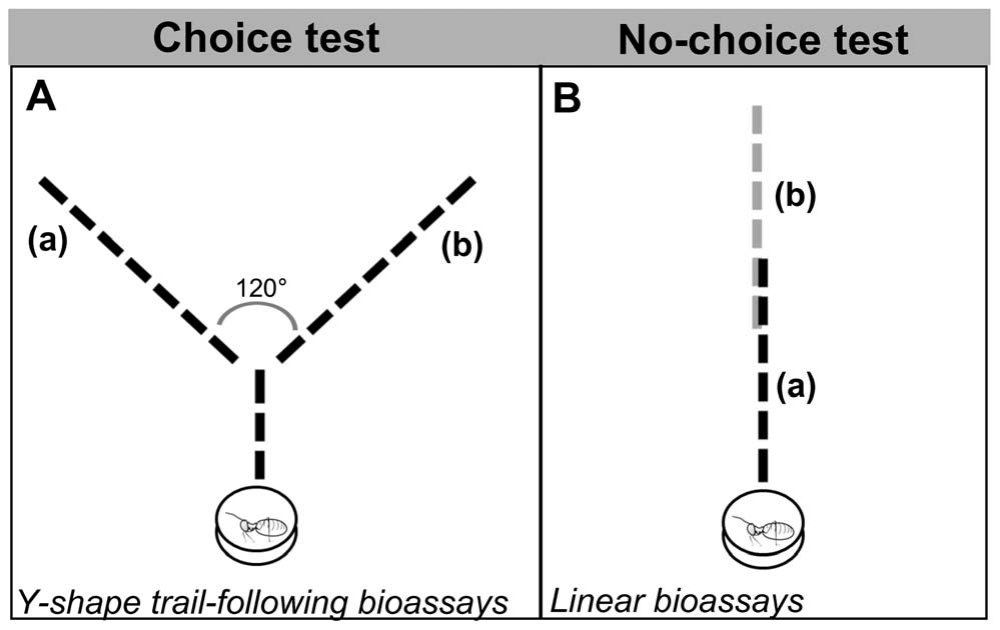
Schematic design of trail-following bioassays: Choice test made with Y-shape trail-following bioassay (A) and no-choice test made with linear bioassay (B). In drawing A, the Y stem was 3° angle between them. In drawing B, the trail consisted of two trails of 6 cm long, made of each extract and overlapping for 2 cm in the centre.

Workers of *C. cyphergaster* and *I. microcerus* were subjected to Y-shape trail-following bioassays (see Fig. 1A), as described in [35] to test their orientation activity to *(i)* their own WBE (in equivalents per cm) or SGE (in equivalents per cm) and *(ii)* standards (neocembrene, dodecatrienol and mixture of neocembrene and dodecatrienol) (concentrations in ng per cm). Equivalent choice tests were also done to compare the orientation activity of SGE and standards in *I. microcerus* workers (see Tab. 1 for an overview of all bioassays).

**Table 1:** Distance followed by *Constrictotermes cyphergaster* and *Inquilinitermes microcerus* workers in Y-shape trail-following bioassays with whole bodies extract (WBE), sternal glands extract (SGE), (3*Z*,6*Z*,8*E*)-dodeca-3,6,8-trien-1-ol (D) and/or neocembrene (N) (n = 30, degrees of freedom = 3, concentration in body or gland equivalent per cm [Eq/cm]).

Tested species	Extract or standard	Concentration	Distance followed (cm)	*F* value	*P* value
*Constrictotermes cyphergaster*	WBE	1 Eq/cm	7.5±0.33 **a**	34.6	0.0006
	SGE	10^−1^ Eq/cm	9.3±0.15 **b**		
	SGE	1 Eq/cm	9.4±0.29 **b**		
*Inquilinitermes microcerus*	WBE	1 Eq/cm	6.3±0.55 **a**	7.01	0.02
	SGE	10^−1^ Eq/cm	8.7±0.43 **b**		
	SGE	1 Eq/cm	8.1±0.30 **b**		
	D	10^−3^ ng/cm	1.5±0.15 **a**	15.92	0.003
	D	10^−2^ ng/cm	5.1±0.58 **b**		
	D	10^−1^ ng/cm	5.3±0.08 **b**		
	D	1 ng/cm	2.5±0.18 **a**		
	N	10^−4^ ng/cm	2.2±0.20 **a**	30.36	< 0.001
	N	10^−3^ ng/cm	2.4±0.10 **a**		
	N	10^−2^ ng/cm	4.9±0.21 **b**		
	N	10^−1^ ng/cm	2.8±0.32 **a**		
	N	1 ng/cm	1.3±0.14 **a**		
	D + N	10^−1^ + 10^−2^ ng/cm	6.0±0.23	159.7	< 0.001
	D + N	10^−2^ + 10^−2^ g/cm	2.9±0.30		

The activity threshold was defined as the minimal concentration eliciting termites to travel over 3 cm on the trail; maximal possible distance was 10 cm (mean ± SE). Hexane was used as a control, and was never followed by termites. Values with the same letters indicate non-significance in Contrast Analyses under Normal distribution.

#### Interspecific trail-following

Interspecific trail-following bioassays were performed to test *(i)* the orientation on trails made with their own extract (conspecific, CS) *vs.* trails made of the other species (heterospecific, HS) and *(ii)* the possible exploitation of *C. cyphergaster* (host) trail-following pheromone by the *I. microcerus* (inquiline) and *vice versa*. Two types of experiments were performed (see Fig. 1): *(i)* choice test (Y-shape trail-following bioassays) and *(ii)* no-choice test (linear trail-following bioassays), as described below.

In the choice test (see Tab. 2 for an overview of all bioassays), two sets of experiments were done: *(i)* CS trail *vs*. HS trail, and *(ii)* CS trail *vs*. mixed trails (MIX; trail made with both species extracts in an equal proportion, mixed before using). The insects were released at the base of the Y-shape scent trail and the distance travelled was measured while noting the chosen Y arm.

**Table 2:** Choice test of *Constrictotermes cyphergaster* or *Inquilinitermes microcerus* workers in Y-shape trail-following bioassays with conspecific (CS), heterospecific (HS), or conspecific and heterospecific mixed (MIX) trails made with 10^−1^ sternal glands extract equivalent per cm (n = 30, degrees of freedom = 3).

Tested species	Set of bioassays	*Chi* value	*P* value
*Constrictotermes cyphergaster*	CS×HS	**1.03**	**<0.001**
	CS×MIX	**0.70**	**0.0002**
*Inquilinitermes microcerus*	CS×HS	**0.72**	**<0.001**
	CS×MIX	**1.03**	**<0.001**

In the no-choice test, two 6-cm trails were laid down from opposite sides of a line. When meeting, such trails would overlap for 2 cm, forming a 10-cm long trail (see Fig. 1B). For each species, two sets of experiments were done: *(i)* CS trail *vs*. HS trail, both made with WBE and *(ii)* CS trail *vs*. HS trail, both made with SGE (see Tab. 3 for an overview of all bioassays). Termites were released at the end of the 10-cm line, starting from the side where their own species' trail was laid down. The distance travelled on this trail was measured while noting, whether the individual followed the trail, left it, or made U-turns to retreat.

**Table 3:** No-choice test of *Constrictotermes cyphergaster* or *Inquilinitermes microcerus* workers in linear trail-following bioassays with conspecific (CS) trail followed by a conspecific trail or a heterospecific (HS) trail made with 10^−1^ whole bodies extract equivalent per cm (WBE) or 10^−1^ sternal glands extract equivalent per cm (SGE) (n = 30, degrees of freedom = 6).

Tested species	Extract	Set of bioassays	Distance followed (cm)	*F* value	*P* value
*Constrictotermes*	WBE	CS then CS	7.6±0.05	0.04	0.8405
*cyphergaster*	WBE	CS then HS	7.5±0.03		
	SGE	CS then CS	9.6±0.01	2.94	0.1615
	SGE	CS then HS	9.3±0.02		
*Inquilinitermes*	WBE	CS then CS	6.4±0.05	**0.70**	**0.0002**
*microcerus*	WBE	CS then HS	5.3±0.03		
	SGE	CS then CS	8.1±0.02	0.39	0.5631
	SGE	CS then HS	8.7±0.01		

Significant effect is in bold.

### Statistical analyses

All analyses utilized Generalized Linear Models (GLM), choosing error distribution according to the nature of the response variable, as described below. Model simplification was done through contrast analyses with *F* tests, combining treatment levels when it did not cause significant (*P<*0.05) changes in the model, as recommended by [36]. Treatments levels are specified below under description of the respective analysis. All analyses were performed in *R* [37], followed by residual analysis to check the suitability of the error distribution and model fitting. To prevent pseudoreplication, values obtained for each of the 10 workers from a given species and colony were collapsed into a single average value. Because such values come from distinct randomly chosen colonies, they stand as true replicates. Similar procedure was used by [38].

To test whether the compounds identified by GCxGC/TOF-MS could elicit behavioural responses in termites, data from the “Intraspecific trail-following” bioassays were analyzed in two separate models for each species. Both models included “distance followed” by the individuals as response variable with a normal error distribution. One of these models included a categorical independent variable (*x-var*) with two levels: “standard” to represent the respective standard compound being tested and “hexane” to serve as a control. The other model included a categorical independent variable (*x-var*) with two levels: “SGE” to represent the extracts of sternal gland and “WBE” to represent whole body extracts. Each of these models was run independently for each bioassay.

To test whether termites would perceive the heterospecific trail, orienting themselves according to it, two models have been built, for each species independently, regarding the experiment “choice-test” of Section “Interspecific trail-following” of Material and Methods. Both models included as response variable “proportion of workers” opting for a given branch of the Y-shape, thus requiring binomial error distribution. The first model included a categorical independent variable (x-var) with two levels: “CS” to represent trails made with extracts of conspecifics and “HS” to represent trails made with extracts of heterospecifics. The second model included a categorical independent variable (x-var) with two levels: “CS” to represent trails made with extracts of conspecifics and “MIX” to represent trails made with a mix of extracts of conspecifics and heterospecifics. Each of these models was run independently for each bioassay.

To test whether termites, in perceiving the trail, would be able to exploit it, two models have been built, for each species independently, regarding the experiment “no-choice test” of Section “Interspecific trail-following” of Material and Methods. One of these models included “distance followed” by the individuals as response variable, thus calling for the use of normal error distribution. This model included a categorical independent variable (x-var) with two levels: “CS-then-CS” to represent treatments where both sides of the 10-cm line contained trails made with extracts of conspecifics and “CS-then-HS” to represent treatments where one side to the line contained trails made with extracts of conspecifics and the other side contained trails made with extracts of heterospecifics. The other model included “proportion of individuals exhibiting U-turns” relative to the total of tested individuals, as its response variable, thus requiring binomial error distribution. Both models were run independently for each bioassay.

## Results

### Anatomy of sternal glands

**Figure 2:**
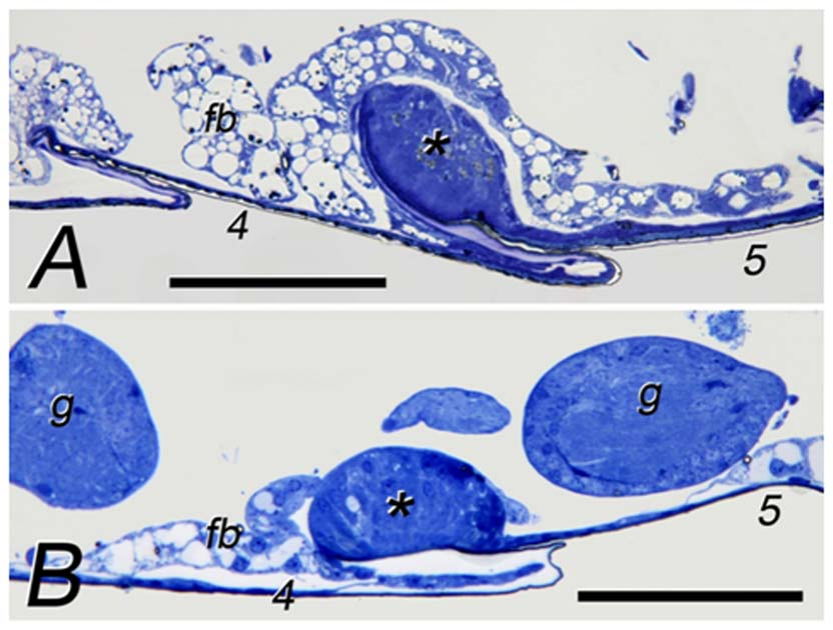
Worker sternal glands of *Constrictotermes cyphergaster* (A) and *Inquilinitermes microcerus* (B). Scale bars represent 100 µm. Numbers mark particular sternites. Asterisks mark sternal glands. Abbreviations: fb - fat body; g - ganglium.

Sternal glands of both species are located on the anterior part of the 5th sternite (Fig. 2). The glands are ovoid in shape and of comparable size (about 80 µm in length and 50 µm in height in both species) but the gland width is slightly larger in *C. cyphergaster* compared to *I. microcerus* (150±7.07 µm *vs.* 120±2.83 µm (mean±SD), respectively) which corresponds to a difference in body sizes: *C. cyphergaster* 4.33±0.41 mm; *I. microcerus* 3.50±0.43 mm (mean±SD).

### Chemical analyses

The GC-MS analysis of extracts of *C. cyphergaster* worker sternal glands revealed the presence of dodecatrienol and neocembrene; both retention indices and MS spectra were identical with those of standards. The presence of these two compounds was already identified in *C. cyphergaster* [27]. The detected amounts per individual were approximately 0.02 ng of dodecatrienol and 1 ng of neocembrene.

In spite of repeated attempts to inject concentrated samples, neither dodecatrienol nor neocembrene or any other known termite trail-following pheromone was detected in WBE or SGE of *I. microcerus* workers.

### Intraspecific trail-following bioassays

#### Trail-following bioassays with WBE and SGE

Both species followed conspecific trails, made either with WBE or SGE. SGE were in both species more efficient in eliciting the trail-following behaviour of workers than WBE (*C. cyphergaster P* = 0.0006; *I. microcerus P* = 0.02; Tab. 1). There was no significant difference between the two concentrations of SGE used (1 and 10^−1^ gland equivalent/cm) (Tab. 1).

#### Trail-following activity of *I. microcerus* with standards

*Inquilinitermes microcerus* workers followed dodecatrienol and neocembrene trails. The highest trail-following activity was reached with 10^−2^ and 10^−1^ ng/cm for dodecatrienol (*P*<0.003; Tab. 1), while only a single concentration of neocembrene (10^−2^ ng/cm) elicited significant trail-following activity (*P*<0.0001; Tab. 1). The highest overall trail-following activity was observed using a mixture of dodecatrienol and neocembrene at concentrations 10^−1^ ng/cm and 10^−2^ ng/cm, respectively (*P* = 0.0002; Tab. 1). Trails made of a mixture of both standards (10^−1^ ng/cm of dodecatrienol and 10^−2^ ng/cm of neocembrene) were significantly more efficient in eliciting the trail-following behaviour of *I. microcerus* workers compared to trails made of each standard alone at the same concentration (*P*<0.02).

The choice test between trails made with *(i)* the mixture of dodecatrienol (10^−1^ ng/cm) and neocembrene (10^−2^ ng/cm), and *(ii)* trails made with SGE (10^−1^ gland equivalent per 1 cm) resulted in a clear preference of *I. microcerus* workers towards the sternal gland extract (*P*<0.0001).

### Interspecific trail-following bioassays

#### Choice test

**Figure 3:**
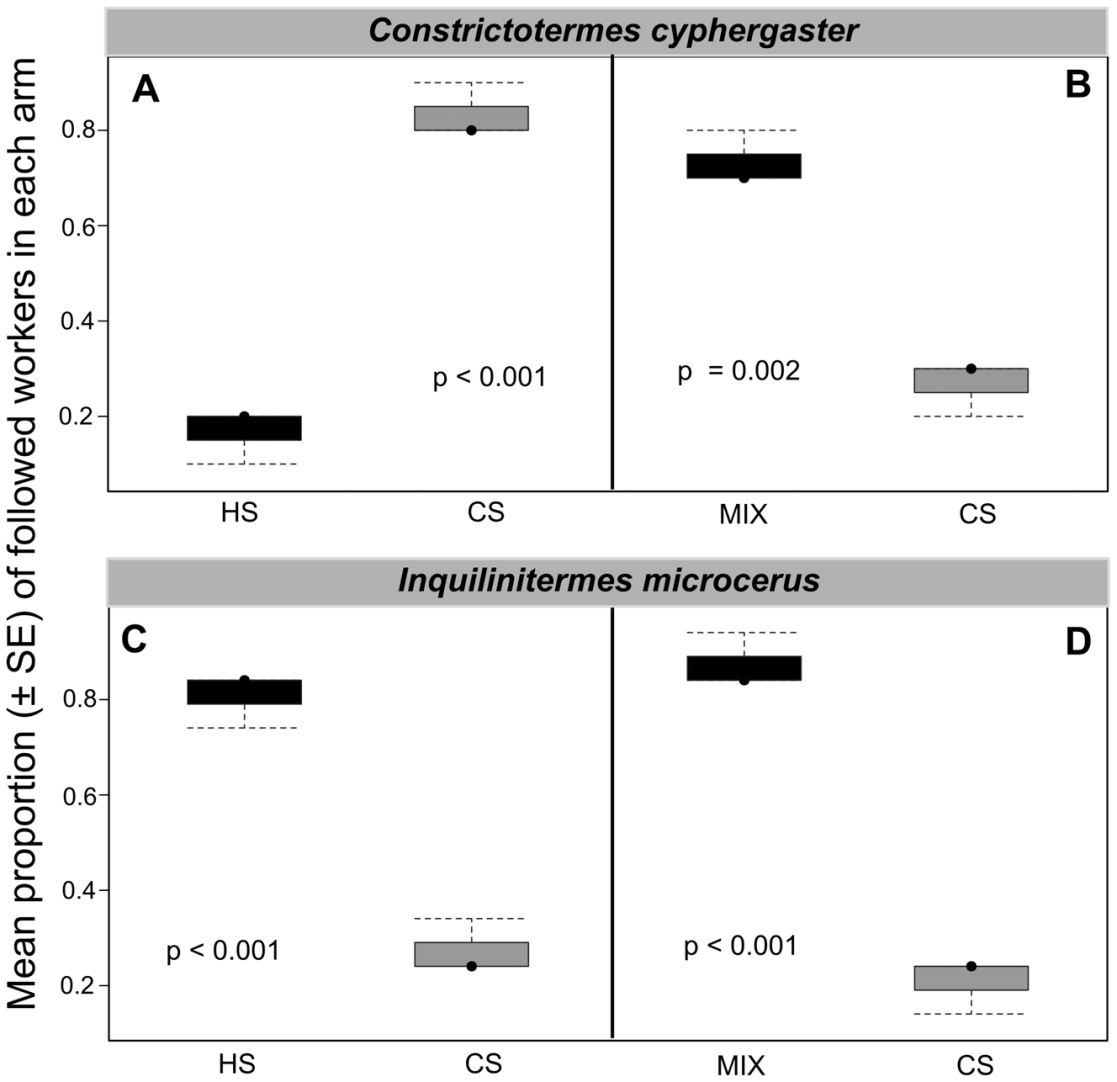
Trail recognition by *Constrictotermes cyphergaster* and its inquiline *Inquilinitermes microcerus*. Each of the four panels depicts a choice test (see Fig. 1A) in which 30 workers (ten from each of three colonies) were exposed to two distinct trails: heterospecific (HS) *versus* conspecfic (CS) in panels A and C; mixed trail (MIX; HS+CS) *versus* conspecific (CS) in panels B and D. In each panel, the vertical axis depicts the mean proportion (± SE) of the number of workers opting for a given arm of the Y-shape. When given the choice between its own trail and that of the other species, both host and inquiline preferred trails of *C. cyphergaster*. If this choice was between own trail and a MIX of both, host and inquiline preferred the MIX trail.f.

In choice tests, the interspecific trail-following bioassays showed that workers of *C. cyphergaster* significantly prefer the CS trail over the HS trail (*P*<0.0001, Tab. 2; Fig. 3A), although they prefer significantly the MIX trail over the CS trail (*P*<0.0001, Tab. 2; Fig. 3B). In contrast, workers of *I. microcerus* always preferred HS trail (*P*<0.0001, Tab. 2; Fig. 3C) or MIX trail (*P*<0.0001, Tab. 2; Fig. 3D) over CS trail.

#### No-choice test

**Figure 4:**
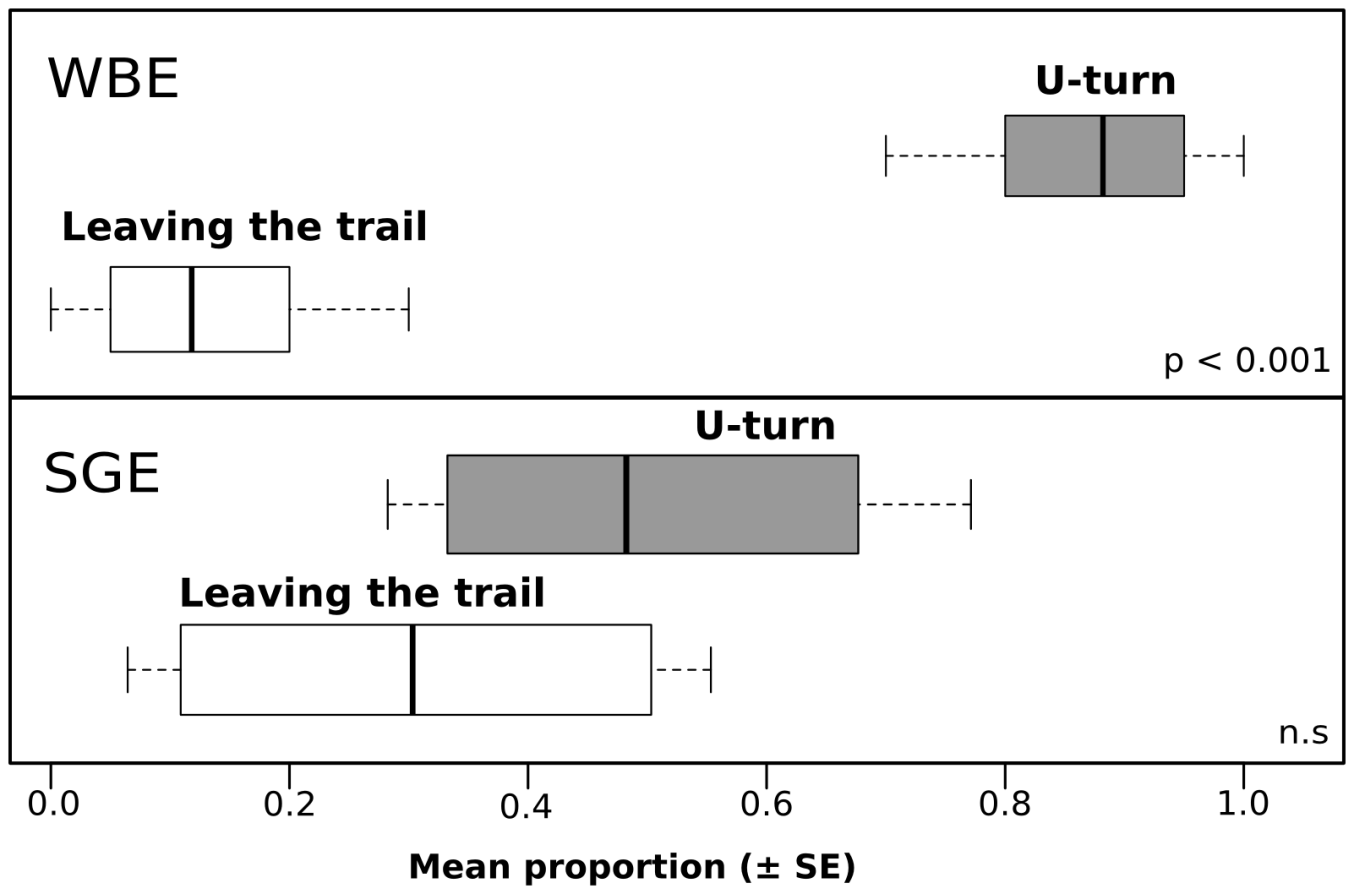
Avoidance of *Constrictotermes cyphergaster* trails by its inquiline *Inquilinitermes microcerus*. Each panel depicts behavioural responses of the inquiline when facing its host trail in a linear bioassay (see Fig. 1B) in which trails were made of whole body extracts (WBE, 10^−1^ whole body equivalent per cm) or sternal gland extracts (SGE, 10^−1^ gland equivalent per cm) of the host. Horizontal axis depicts the mean proportion (± SE) of the number of workers leaving the trail or making U-turns when perceiving the host's trail. *Inquilinitermes microcerus* clearly avoided the WBE host trails making U-turns but do not exhibit such avoidance if the trail was made of SGE.

In no-choice tests, workers of *C. cyphergaster* were able to follow the same distance on both CS and HS trails made of WBE (*P* = 0.84, Tab. 3), while *I. microcerus* followed their CS trail for longer distances compared to HS trail (*P* = 0.0002, Tab. 3). When workers of *I. microcerus* reached the HS trail made of WBE, they made a U-turn and retreated back to the release chamber. This behaviour was performed by *I. microcerus* significantly more often than by *C. cyphergaster* (*P*<0.001; Fig. 4).

However, the same tests performed with SGE instead of WBE resulted in the same distance travelled on the CS and the HS trail for both species studied (*P* = 0.16 for *C. cyphergaster*; *P* = 0.56 for *I. microcerus*; Tab. 3). Moreover, the frequency of U-turn after encountering the trail of the other species did not differ (*P* = 0.32).

## Discussion

### Trail-following pheromones and their activities

Based on trail-following bioassays and chemical analyses, we confirmed that neocembrene and dodecatrienol are the major compounds of the trail-following pheromone of *C. cyphergaster* [27] and we estimated the quantity of both compounds in the sternal glands of workers to be approximately 1 ng and 0.02 ng, respectively. Despite several attempts to confirm the nature of the trail-following pheromone in *I. microcerus* by measuring sternal glands extracts concentrated up to 200 equivalents in one single injection, the chemical composition could not been confirmed. Theoretically, taking into account the low diversity of trail-following pheromones in termites and their distribution through termite species (for a comprehensive historical account, see [22], [23]), three possibilities were expected for the chemical nature of *I. microcerus* trail-following pheromone: *(i)* dodecatrienol alone, *(ii)* mixture of dodecatrienol and neocembrene and less likely but not impossible *(iii)* either of the above plus a minor and unknown compound. Based on our trail-following bioassays, it was clear that dodecatrienol alone is not the trail-following pheromone in *I. microcerus*, since this compound did not elicit termites to follow a long distance on the trail in comparison to a mixture of dodecatrienol and neocembrene (see Tab. 1). It is, thus, likely that when it comes to trail-following pheromones, *I. microcerus* shares the same compounds as *C. cyphergaster*. Minor and unknown compounds may also be present since *I. microcerus* workers clearly preferred their sternal gland extracts over a mixture of dodecatrienol and neocembrene standards in trail-following bioassays (*P*<0.0001).

Identification of termite trail-following pheromones is known to be difficult due to minute pheromone quantities [39]. More recent techniques like GC-EAD have proven to be useful in such study by highlighting some minor compounds that traditional techniques of chemical analyses could not identify [26]. However, even GC-EAD was not successful in identifying dodecatrienol and neocembrene in *I. microcerus* in part due to extremely short (compared to other termites) lifespan of the antennae isolated from workers. Neocembrene is a non-polar diterpene which is usually easily detected by GCxGC/TOF-MS. The absence of detection of this compound in *I. microcerus* samples is surprising given the results of the behavioural experiments. The amount of neocembrene might be of a few picograms only, which is just below the detection limit. Because of the polarity of dodecatrienol causing a tailing peak, the detection limit of this compound is much higher, between 0.1 and 1 ng/µl, while the expected amount is significantly lower.

Interestingly, trail-following pheromones consisting of both neocembrene and dodecatrienol seem to have evolved several times independently, since they have been identified in all Nasutitermitinae (Termitidae) studied so far (present study, [27]), as well as in the *Amitermes evuncifer* (Termitidae: Termitinae) [28] and in *Prorhinotermes* spp. (Rhinotermitidae) [26]. Alternatively, trail-following pheromones consisting of neocembrene and dodecatrienol may represent synapomorphy of a clade comprising Rhinotermitidae (+ Serritermitidae) + Termitidae [25], [40]–[42], but this hypothesis would require numerous secondary losses of one component (neocembrene) or both components (for distribution of particular compounds in all taxa see [22], [23]).

*Inquilinitermes microcerus* is found only in *C. cyphergaster* nests, but predominantly in parts rarely visited by *C. cyphergaster* (although located in general in the colony centre, representing perhaps the oldest nest parts). Such places seem to represent deposits of waste (hosts faeces and dead bodies) and it is not used by the host colony [17]. *Inquilinitermes microcerus* has never been observed outside the host nest and existing studies indicate that *I. microcerus* workers feed predominantly on the dark grey (abandoned) parts of the nest [17]–[20]. Thus, although *I. microcerus* is a clear example of an inquiline as defined by [5], it is also similar to ‘one-piece’ life type termites (e.g. Kalotermitidae). Its ecology may explain the low amount (hundreds of picograms) of trail-following pheromone present in sternal glands of *I. microcerus*: ‘one-piece’ life type termites as well as obligate inquilines do not need high amounts of trail-following pheromone due to the limited space crossed [35]. The confined domain of these termites contrasts with the ‘separate’ or ‘central’ life type termites, in which workers must use considerable amounts of a trail-following pheromone in order to reach their foraging sites and mark their way back to the nest [43]. Although ‘one-piece’ life type termites and inquilines are capable of laying trails, the efficiency of trail-following pheromones might not be crucial for colony success since colonies spend their whole life in the same place using it as nest and food source. Thus, the role of the sternal gland secretion in ‘one-piece’ termites is considered more like a recruitment signal to lead termites to sources of disturbance, rather than an orientation signal [26], [44], [45]. Nevertheless, it was recently observed that under certain conditions, ‘one-piece’ termites were able to move from a piece of wood to another and to use connecting runways requiring the utilization of trail-following pheromones as orientation [22], [46], [47].

It is known that an epigeous structure initially houses the colony of *C. cyphergaster* while an arboreal nest is built only after the population exceeds a certain number [31]. The back and forth movement of workers from the initial nest to the new one under construction clearly involves the use of a trail-pheromone. It has also been showed that *I. microcerus* preferably occupies *C. cyphergaster* arboreal nests of rather larger size (≥13 L.; [14]) but the mechanisms of the *C. cyphergaster* nest location by *I. microcerus* remain mysterious. It seems probable that the invasion of the inquiline starts by penetration of an *I. microcerus* dealate pair which uses visual cues at long distance to identify a suitable nest, but also chemical cues (including trail-following pheromone) perceived at short distance. The identical chemical nature of the trail-following pheromone of both species may help imagoes of *I. microcerus* to find and enter the nest of their hosts. Trail following by cohabitants to migrate between nests was already observed in lycaenid caterpillars and their host ants (see [48]). Some staphylinid termitophiles are also able to follow trails laid by their termite hosts, hypothetically not only to locate their resource (the termites), but also to locate their few conspecifics [49].

### Interspecific trail following: How does trail following shape the relationships between *C. cyphergater* and *I. microcerus*?

This study showed that *I. microcerus* workers always preferred the trails made of *C. cyphergaster* extracts (Fig. 3C) or the MIX trails (Fig. 3D) rather than trails made of their own extracts. Conversely, *C. cyphergaster* workers prefer trails made by their own extracts (Fig. 3A) or by both extracts (Fig. 3B) compared to trails of *I. microcerus* extracts. These results may be due to the quantity of pheromone being higher in *C. cyphergaster* than in *I. microcerus*. These behavioural results might be linked to the size of the sternal gland being larger in *C. cyphergaster* than in *I. microcerus*. We cannot exclude that minor compounds present in the sternal gland secretion of *C. cyphergaster* may explain these trail preferences even though we did not detect any relevant compounds.

The U-turn behaviour performed by *I. microcerus* workers after detecting WBE of the host (Fig. 4) indicates perception and active avoidance of the host smell, probably due to presence of repellent compound(s) secreted by *C. cyphergaster* workers for their own protection during foraging in the open air. This hypothetical compound is obviously not present in the sternal gland secretion, and may originate either from the enlarged mandibular glands of workers [50] or from the faeces of *C. cyphergaster* workers, which are known to be laid onto their trails [51], [52]. Moreover, powerful repellents are expected in open-air foraging termites to minimize predation upon them. Although soldiers play the prime role in defence and their high numbers (44% during the dry season and 33% during the wet season – see [29]) explain *Constrictotermes* success, we may also expect the appreciable effect of repellents used to avoid predation. Chemical repellence has been already reported as effective against mammalian termite predators [53].

The strategy used by the inquiline is to build its nest inside the host nest with no connection between the two nests thereby minimizing probable conflicts. Due to its comparable body size, the host could probably penetrate inquiline gallery system and kill all inhabitants based on its numerical dominance, if the inquiline would not able to detect gaps in the nest by using the trail-following pheromone of the host. *Inquilinitermes microcerus* can rely on the chemical cues of *C. cyphergaster* to inhabit its nest without risk of confrontation. Another possibility could be the use by *I. microcerus*, of *C. cyphergaster* trail-following pheromone as an indication of opportunities or threats. A low concentration of the trail-following pheromone due to a breach that needs to be sealed by the inquiline may be considered as a low level threat and may trigger investigation for *I. microcerus*. In contrast, a high concentration encountered when an important breach is created into the nest or when the inquiline digs into chambers with relatively fresh and potentially infectious corpses, may induce a quick retreat in *I. microcerus*. This could explain the different results between whole body and sternal gland extracts in Fig. 4.

In short, our results seem consistent with the hypothesis that trail-following pheromones may shape the cohabitation of *C. cyphergaster* and its guest *I. microcerus*. This is the first study evaluating chemical communication between two closely associated termite species. It seems evident that the inquiline is able to use the host's chemical cues to evade detection within the nest. While strictly in line with previous findings that cohabitation by this same pair of species is eased by the use of distinct diets [14], our results reinforce the idea that inquilinism by *I. microcerus* is based on conflict-avoidance strategies.
